# Reconsidering thought suppression and ironic processing: implications for clinical treatment of traumatic memories

**DOI:** 10.3389/fpsyg.2024.1496134

**Published:** 2024-12-11

**Authors:** Zulkayda Mamat, Daniel A. Levy, Peter J. Bayley

**Affiliations:** ^1^War Related Illness and Injury Study Center, VA Palo Alto Health Care System, Palo Alto, CA, United States; ^2^Department of Psychiatry and Behavioral Sciences, Stanford University School of Medicine, Stanford, CA, United States; ^3^Department of Psychology, Palo Alto University, Palo Alto, CA, United States; ^4^Baruch Ivcher School of Psychology, Reichman University, Herzliya, Israel

**Keywords:** ironic process theory, ironic rebound effect, thought suppression, memory suppression, cognitive control, direct suppression, unwanted thoughts, intrusive thoughts

## Introduction

It is not difficult to see why suppression of traumatic or otherwise unwanted memories is unpopular as a therapeutic tool. Already under the shadow of reservations regarding psychoanalytic repression theory, suppression was further devalued by its association with claims regarding repressed recovered memories (Loftus, 1993). Such claims stated that emotional disorders characterized by dissociative symptoms could be treated through the recovery of repressed memories using tools including suggestion, hypnosis, and dream interpretation. Opponents of this approach asserted that techniques ostensibly intended to allow accurate retrieval of suppressed memories of childhood abuse actually led to the formation of false memories, resulting in accusations directed toward innocent parties (Lindsay and Read, 1994). These “memory wars” of the 1990s led to skepticism not only regarding recovery but regarding the possibility of memory repression which continues to affect research and society today (Battista et al., 2023). Furthermore, cognitive behavioral therapy, developed in the 1960s, saw memory suppression as antithetical to its core focus on relieving the effects of traumatic memories through reappraisal. It seems intuitive that resolving traumatic memories is better than suppressing them. It was not until the 1980s that experimental evidence, under the banner of ironic processing theory (IPT), was brought forward to rationalize this state of affairs.

A series of studies, widely referred to as the “white bear” experiments, was used to demonstrate a supposed major problem with suppression: the more one tries to suppress thoughts, the more likely they are to return. IPT explains this effect in terms of two mental processes required to control the contents of consciousness: an intentional operating control process and an unconscious monitoring process (Wegner, 1994). The latter process, scanning memories and environmental cues in its vigilant effort to detect violations of the person's intent, ironically brings to mind precisely the contents which the first system is trying to avoid. These conclusions were extended beyond simple thought suppression to claims regarding suppression of emotions, memory, interpersonal processes, psychophysiological reactions, and psychopathology (Wenzlaff and Wegner, 2000).

## Alternative explanations for the ironic rebound effect

Do those ironic processing studies' findings justify the rejection of suppression as a therapeutic tool? All human-subject experiments depend on the participant's understanding of the instructions as intended by the experimenter. In the white bear experiment (Wegner et al., 1987), the participants were instructed to “…try not to think of a white bear. Every time you say, “white bear” or have “white bear” come to mind, though, please ring the bell on the table before you.” While seemingly straightforward, a closer look at these instructions reveals a number of issues which challenge the generalizability of the conclusions based on them.

### Ambiguities in thought suppression strategies

Firstly, the mode of suppression intended in the phrase “*try not to think”* is unclear. It is possible not to think about something using a variety of strategies: direct suppression (e.g., momentarily blocking out all thoughts from coming into mind), thought substitution (e.g., thinking of a pink elephant), self-distraction (e.g., repeating a tune in one's mind), and mind-wandering (e.g., daydreaming about dinner plans). Each of these strategies vary in level of effort required and in their effectiveness in enabling the participant to “not think.” Additionally, there were no follow-up measures to check whether participants were indeed continually engaging in an effortful and intentional suppression (i.e., direct suppression) that could qualify as mental control. Furthermore, the phrase “*every time”* suggests that it will most certainly occur, which may elicit expectations of failure by the participant.

### Task switching as a confound in load-induced suppression

Variations on the white bear experiments illustrate how the specific parameters of the experiments might be responsible for the observed “ironic effect.” Versions of the white bear experiment under cognitive load (an additional effortful task during the trials, e.g., to recall a previously learned number) showed evidence of “a load-induced surge of suppression-related material,” ostensibly due to the added cognitive demand disabling thought suppression (Wenzlaff and Wegner, 2000). A closer look reveals that the additional load involves task switching, which can render suppression efforts ineffective in four different ways. Firstly, task switching may bring about increased accessibility under load by enhancing memory for task-irrelevant content while impairing task-relevant information (Richter and Yeung, 2012). Secondly, when cognitive load triggers task switching, it disrupts the repetitions needed for effective suppression via some of the abovementioned strategies, leading to increased intrusions and hyperaccessibility of suppressed information during testing. Thirdly, task switching may directly inhibit suppression. In the dual task paradigm, the suppression trial procedure involves three tasks: (A) suppressing a target word, (B) listening to related or unrelated words, and (C) providing word associations. When switching from task A to B, the inhibition of task A occurs (Mayr and Keele, 2000). This inhibition persists until the task switches back to A (A → B → C → A), requiring the inhibition to be overcome, resulting in deinhibition costs like slower response times and increased errors (Chen et al., 2022). Thus, it is quite possible that maintaining suppression under cognitive load that does not require task switching would not be susceptible to rebound of repression target material.

There are similar issues with another experiment adduced to illustrate ironic rebound of suppressed thoughts, in which participants were asked to recall either a sad or happy life event and write down their reminiscing thoughts of the event while *trying to not be sad/happy* or *trying to be sad/happy* (and reported to have been “clearly unsuccessful” for the former, “marginally unsuccessful” for the latter) (Wegner et al., 1993). In both these types of manipulations, it is asserted that cognitive load is “intrusion-promoting.” However, intrusion reports may increase because: (1) the retrieval process initiated by the cognitive load task temporarily brings back more recent items into working memory, and (2) the nature of the suppression process requires (temporary) intrusions of the to-be-suppressed material in order for it to be suppressed for an extended period. In other words, the suppression process had not been given its proper time course to be completed such that intrusion frequency was assessed at its natural peak. This is similar to the second effect of task switching described above as an alternative explanation for the observed load-induced hyperaccessibility. Ironically, Wenzlaff and Wegner (2000) dismissed the goal interruption theory—that an unfulfilled need to complete the goal of thought suppression is the cause of rebound—and preferred the ironic rebound theory because of the former's inability “to account for the return of intrusive thoughts during suppression.”

Recent studies challenge the prevailing dogma that suppression leads to increased intrusion into awareness. For instance, experiments conducted by Anderson and Green (2001) demonstrate that actively attempting to suppress unwanted memories can induce forgetting [referred to as the suppression induced forgetting (SIF) effect], contradicting the notion of a rebound effect. Since then, research has provided a wealth of insights into memory suppression, from neural mechanisms underlying thought suppression (Anderson and Hulbert, 2021; Apšvalka et al., 2022) to weakening of not only explicit but implicit expressions of memory through suppression practice (Taubenfeld et al., 2019) to the potential of memory suppression as a therapeutic approach for addictive behaviors (de Almeida-Antunes et al., 2024; Noël, 2024) to a recent demonstration of suppression training as a valid therapeutic approach to alleviate anxiety, especially in the most vulnerable populations (Mamat and Anderson, 2023). Having been demonstrated to be a tool with many benefits, suppression cannot be dismissed.

Putting aside the problematic conclusions of the IPT approach that have hindered a full appreciation of the application of suppression in improving wellbeing, it is important to acknowledge that, like any intervention, suppression is not going to be effective in all circumstances across all populations. For instance, some studies suggest that depressive symptoms may impair inhibitory control over memory retrieval, potentially contributing to depression development (Joormann et al., 2009; Sacchet et al., 2017). However, therapeutic training might enable people contending with depression to better exercise effective thought control. Research on suppression training (Mamat and Anderson, 2023) certainly suggests that suppression ability is dynamic—it can be taught, learned, and improved upon, even in people with clinical depressive symptoms. The effectiveness of thought suppression is typically measured through the SIF effect. This raises critical questions about the causal relationship between SIF and mental health disorders. Do individuals with certain disorders exhibit a low SIF effect because their memory suppression ability has been weakened by the disorder (and is therefore potentially restorable), or is a low SIF effect a marker of inherently deficient suppressive abilities, leading to disorder severity? This distinction is vital for clinicians, as therapeutic approaches may vary significantly depending on whether the focus is on strengthening an inherently weak ability, addressing a skill diminished by the disorder, or enhancing an underutilized capacity. Given the variability in reported effect sizes of SIF, replication studies are essential to establish their reliability in clinical populations. Endeavors such as multi-site replication efforts of the SIF effect (Fawcett et al., 2023) are needed to accurately determine effect sizes and refine therapeutic applications, as they may reveal individual differences in response to thought suppression techniques (Nardo and Anderson, 2024). Ultimately, while thought suppression holds potential for therapeutic use, careful consideration of methodological variations and long-term efficacy is essential for its successful integration into clinical practice.

## Discussion

Research indicates that memory suppression may aid post-traumatic adaptation in PTSD patients (Mary et al., 2020). Similarly, participants meeting provisional PTSD diagnosis experienced sustained mental health benefits 3 months after memory suppression training, but only when suppressing negative rather than neutral content (Mamat and Anderson, 2023). Clinical protocols with multiple sessions of suppression may yield stronger effects than in lab studies, where only single sessions are typically tested. Indeed, improvement in mental health was observed after repeated suppression training on pandemic-related intrusive thoughts, with over 80% reporting long-term use of the direct suppression technique in their own lives outside of the laboratory context, even among those with PTSD symptoms (Mamat and Anderson, 2023). Therefore, especially given the evidence regarding its potential to be learned by vulnerable populations and its subsequent beneficial impact on mental state (Mamat and Anderson, 2023), our intention is to open the possibility that memory suppression may be available as an approach in the wellness toolkit, to be selected when appropriate. Furthermore, given the increasing popularity of mindfulness-based interventions, perhaps it is also apt to ask: is not the “letting go of thoughts” in mindfulness a form of directly suppressing the representation of those thoughts from manifesting further in consciousness? The relationship between mindfulness practice and inhibitory control regions of the brain has been established (Bailey et al., 2019), inviting further exploration into investigating the nuances of shared mechanism for control of thoughts.

Memory suppression shows great promise as an adjunctive tool in clinical settings. Research demonstrating the possibility of suppression of future-oriented fears and worries (Benoit et al., 2016; Mamat and Anderson, 2023) suggests that the technique might help veterans and others who exhibit PTSD, OCD, and phobias to suppress intrusive memories or thoughts. Future research directions may include investigating the neural adaptations associated with memory suppression training, which could inform the development of targeted interventions using techniques like transcranial magnetic stimulation (TMS). Additionally, exploring the integration of memory suppression methods with existing treatment modalities, such as neurofeedback, could lead to more comprehensive and effective therapeutic strategies for various mental health disorders. Empirical investigation of these possibilities is warranted by extant findings. Looking ahead, we suggest the time is right for thought suppression to be more seriously considered as a therapeutic tool for mental health.
